# Effect of *Saengshik* Supplementation on the Gut Microbial Composition of Healthy Korean Adults: A Single-Group Pilot Study

**DOI:** 10.3389/fnut.2021.743620

**Published:** 2021-10-22

**Authors:** Ji-Hee Shin, Yong Ju Ahn, Won-Hyong Chung, Mi Young Lim, Seungpyo Hong, Joong-Hark Kim, Mi Houn Park, Young-Do Nam

**Affiliations:** ^1^Research Group of Healthcare, Korea Food Research Institute, Wanju-gun, South Korea; ^2^Theragen Bio Co., Ltd., Seongnam-si, South Korea; ^3^Department of Food Biotechnology, Korea University of Science and Technology, Daejeon, South Korea; ^4^Erom R&D Center, Erom Co., Ltd., Chuncheon-si, South Korea; ^5^Department of Medical Biotechnology, College of Biomedical Science, Kangwon National University, Chuncheon-si, South Korea

**Keywords:** gut microbiota, raw-food formula, plant-based dietary supplement, enterotype, *Saengshik*

## Abstract

*Saengshik* is a type of meal-replacement product or dietary supplement comprising an uncooked and dried plant-based food mixture with various health-promoting properties, such as antidiabetic, anti-dyslipidemic, antioxidant, and anticancer properties. Although these properties are considered attributable to the various bioactive components absorbed through the intestine and its remolding effect on intestinal microorganisms, the effect of *Saengshik* supplementation on gut microbiota profiles has not yet been studied. In this study, we investigated the effect of *Saengshik* administration on the composition of gut microbiota. This single-group design trial was conducted on 102 healthy men and women who received 40 g/day of *Saengshik* powder for 8 weeks, during which stool samples were collected at two fixed time points (baseline and the endpoint) for gut microbiota-profiling analysis. We observed a significant decrease in the α-diversity of gut microbiota after *Saengshik* consumption (*P* < 0.05), with significant changes identified in the composition of major microbial taxa, such as *Bacteroidetes* (*P* < 0.0001), *Proteobacteria, Actinobacteria*, and *Verrucomicrobia* (*P* < 0.0001). Notably, the gut microbial response was related to the inter-individual variability of habitual dietary intake and enterotype at baseline. To the best of our knowledge, this is the first study investigating the effects of *Saengshik* intake on changes in gut microbiota, with the results suggesting that individual habitual diet patterns and gut microbial shapes should be considered key aspects in *Saengshik*-mediated health-promotion effects.

## Introduction

According to the Korea Food Standards Codex*, Saengshik* refers to a product processed from plant- or animal-derived materials by drying and can be consumed with beverages, such as water and milk (1). Commercially-available *Saengshik* mainly comprises 30–60 types of whole grains, vegetables, seaweeds, mushrooms, and other minor plant-based ingredients. Due to the process of freeze-drying or low-temperature drying of its ingredients, *Saengshik* preserves various nutrients, including minerals, vitamins, and phytochemicals in raw materials (2–9). Additionally, *Saengshik* consumption has increased based on its advantage of allowing convenient consumption of various plant-derived raw materials.

To date, several studies have reported the beneficial role of *Saengshik*. Given that *Saengshik* is generally a low-calorie meal replacement, anti-obesity effects have been reported through weight-loss studies in obese adults (2, 3). *Saengshik* also exhibits various bioactive functions, including antidiabetic (4, 5), anti-dyslipidemic, antioxidant (4, 6), and anticancer (7, 8) activities. Furthermore, Shin et al. (9) reported that *Seangshik* supplementation attenuated colitis in murine models of inflammatory bowel disease. These beneficial effects might be attributed to the functional ingredients of plant-based foods, such as phytochemicals, minerals, vitamins, and dietary fibers. However, despite evidence of a link between *Saengshik* consumption and health benefits, no intervention studies have assessed its modulation of the gut microbiome.

The role of gut microbiota cannot be overlooked in acquiring an understanding of the physiological effects of specific food components, because microorganisms are an important factor that influences nutrient utilization. The human gut harbors a vast community of microbes that include ~100 trillion bacterial cells, with these microorganisms playing pivotal roles in human health improvement, as well as the pathogenesis of diseases (10, 11). Diet is a major external factor in the variability of gut microbiota composition (12). Specifically, plant bioactive compounds, such as polyphenols and fibers, are transformed by gut microbiota into a bioactive form responsible for human health effects (13, 14). Thus, plant-based dietary modulation of the gut microbiome has been continuously reported in human and animal studies (15–18). Furthermore, emerging evidence suggests that discrepancies in gut microbiota responses to dietary interventions among individuals might be related to differences in both baseline gut microbiota composition and habitual dietary intake (19–21). Individual baseline composition of intestinal microflora is referred to as an enterotype, with this used to classify gut microbiota according to the proportion of dominant bacteria, such as *Bacteroides, Prevotella*, and *Ruminococcus*. A previous study reported that enterotypes are generally affected by individual long-term diet patterns, *Bacteroides*-dominated enterotypes associated with animal fat and protein intake, and *Prevotella*-dominated enterotypes linked to a carbohydrate-rich diet (22). Therefore, individual gut microbial responses to *Saengshik* intake could be influenced by indigenous gut microbiota and habitual dietary intake.

The objectives of this study were to confirm changes in intestinal microflora after *Saengshik* intake and verify the hypothesis that these changes might be related to the status of habitual dietary intake and baseline gut microbiota composition.

## Materials and Methods

### Study Participants

The participants were recruited using posters at Theragen Bio (Seongnam, Gyeonggi, South Korea). The eligible subjects were healthy men and women between 20 and 65 years of age. Individuals who consumed pro-, pre-, or antibiotics within 3 months before the study were excluded. All participants provided written informed consent, and the study protocol was approved by the Institutional Review Board (IRB) of Theragen Bio (IRB No. 700062-20180905-JR-005-01).

### Preparation of the Experimental Formula

*Saengshik* (Eromplus Cellfood Goodmorning Saengshik Premium) was provided by Erom Co., Ltd. (Seongnam, Gyeonggi, South Korea) and prepared by mixing freeze-dried and powdered ingredients and transferring them into plastic packaging. The formula contained grains, beans, vegetables, fruits, mushrooms, seaweed, and other diverse raw materials (Supplementary Table 1). The nutrient contents were measured at SGS Korea Co., Ltd. (Uiwang-si, Gyeonggi-do, Korea) and are summarized in Supplementary Table 2.

### Experimental Design and Intervention

This study was a single-group trial conducted at Theragen Bio from October 2018 to February 2019. On day 1 of subject arrival, they were provided enough *Saengshik* powder in 40-g packages and enough for 8 weeks of consumption. Subjects were instructed to consume 40 g of *Saengshik* powder with 100–150 mL of water daily over the 8-week treatment period. Although not assigned a time to eat *Saengshik*, subjects were asked to report whether they ate *Saengshik* daily. After 8 weeks, subjects that failed consume *Saengshik* more than once were excluded from the analysis due to poor compliance. Additionally, all participants were instructed to maintain their regular diet patterns and avoid any pro- and prebiotic supplements during the study period. Fecal samples from the participants were collected using a commercial fecal sampling kit (OMNIgene GUT; OMR-200; DNA Genotek, ON, Canada) at two fixed time points (at baseline and the study endpoint) for gut microbiota-profiling analysis (Supplementary Figure 1). The instructions specified in the stool sampling kit were explained, with each subject instructed to collect a specific amount of stool.

### Habitual Dietary Assessment

Habitual dietary intake was assessed at baseline using the food frequency questionnaire (FFQ) method, which was developed and validated by the Korea Centers for Disease Control and Prevention (23). The FFQ included 103 food or beverage items with three serving sizes and nine intake-frequency questions, with the survey used to assess the items consumed over the previous year. The validity and reproducibility of the FFQ has previously been verified (23). According to the food intake measured by the FFQ, the amount of calories and nutrients consumed daily by each individual was calculated and corrected to a level of 1000 kcal for use in creating subgroups based on nutrient intake.

### 16S rRNA Sequencing and Data Analysis

The metagenomic DNA of each stool sample was extracted with a QIAcube instrument using a QIAamp DNA stool mini kit (Qiagen, Hilden, Germany) according to manufacturer instructions along with an additional bead-beating procedure. Briefly, 250 aliquots of stool samples were transferred to a tube together with 1.2 mL of ASL lysis buffer and 0.3 g of sterile 0.1 mm zirconia beads (BioSpec, Bartlesville, OK, USA) and homogenized twice at 30 Hz for 1 min on the TissueLyser system (Qiagen) (24). The third to fourth hypervariable region (V3–V4) of the 16S rRNA gene from the extracted DNA sample was amplified and sequenced using the Illumina MiSeq 2 ×300 System (Illumina, San Diego, CA, USA).

The DADA2 pipeline (25) of the QIIME2 package (v.2019.1; https://qiime2.org) (26) was used to generate unique sequence variants by filtering low-quality sequences and chimeras. Taxonomy was assigned using a pre-trained Naïve Bayes classifier based on the Greengenes 13_8 (27) 99% operational taxonomic units (OTUs), which had been trimmed to include the V3–V4 region. Chimeric reads were filtered from the sequencing data using UCHIME software (28). After excluding samples with low sequencing depths (<10,000 reads), the gut microbial compositions were analyzed in 66 paired samples (29 men and 37 women) and compared before and after *Saengshik* supplementation. The relative abundance of bacterial genera was calculated from sequencing data at baseline, with this data used to establish the enterotype of the subject.

### Statistical Analyses

Statistical analysis was performed using GraphPad Prism software (v.8.0.1; GraphPad Software Inc., San Diego, CA, USA) and R studio (v.1.1.447) (29). Differences in genera and phyla between before and after *Saengshik* supplementation were assessed using a Wilcox test in R studio using the “wilcox.test (paired=TRUE)” function from the “stats” R package. Significant changes in bacterial taxa between groups were calculated using a two-tailed Mann–Whitney *U* test (GraphPad Prism v.8.0). To cluster the gut microbial community of each subject, enterotype assignment was performed based on a published tutorial (http://enterotype.embl.de/enterotypes.html) (22). Briefly, the 66 samples were clustered using the PAM (partitioning around medoids) clustering algorithm in the R package “cluster,” and the optimal number of clusters was chosen based on the Calinski–Harabasz index using the “clusterSim” R-package. The result of enterotype clustering was visualized in a principal component analysis plot using the R package “ade4.” The level of statistical significance was set at *P* < 0.05.

## Results

### Baseline Characteristics

**Figure 1:**
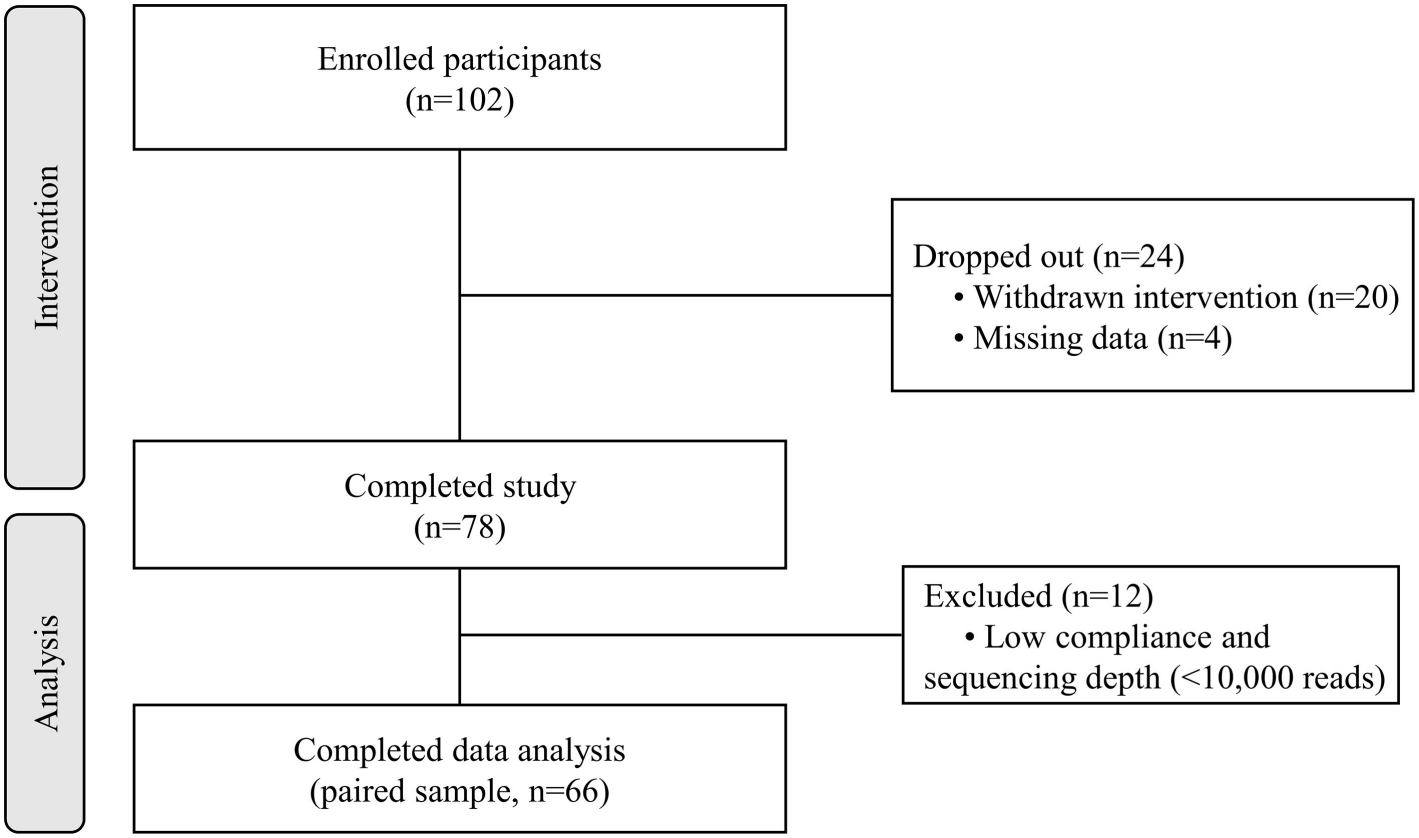
Flow diagram of the study.

A total of 102 individuals who were assessed for eligibility were included in this study, with 11 participants withdrawing before the end of the study. After an 8-week treatment period, we performed 16S rRNA sequencing of fecal samples before and after intervention from 78 participants that completed the study. In total, 12 subjects (15.4%) withdrew from the data analysis owing to an insufficient number of sequencing reads (<10,000 reads) and low compliance. Therefore, the gut microbial composition of 66 participants at baseline was compared with that at post-intervention, as presented in the schematic procedure of the intervention study (Figure 1). Table 1 shows the daily energy and nutrient intake of participants at baseline. At baseline, participants demonstrated a lower intake of energy, carbohydrates, proteins, fats, vitamin B_2_, folate, calcium, phosphorus, potassium, iron, and zinc than that of an average Korean, although intake of vitamins A and C were higher than that of the average Korean. Daily intake of vitamin B_1_ and niacin were similar to the Korean average (Table 1). The baseline characteristics of the participants are shown in Table 2. The 66 participants comprised 29 men and 37 women, with a mean age of 40.89 ± 9.94 years.

**Table 1:** The nutrient intake (g/day) of participants at baseline.

	**All**
	**(*n* = 66)**
Energy (Kcal)	1560.25 ± 703.22
Carbohydrate (g/d)	250.1 ± 128.40
Protein (g/d)	55.93 ± 22.59
Fat (g/d)	36.26 ± 18.48
Cholesterol (mg/d)	206.2 ± 102.40
Fiber (g/d)	4.237 ± 2.93
Vitamin B1 (mg/d)	1.035 ± 0.51
Vitamin B2 (mg/d)	0.8499 ± 0.38
Vitamin B6 (mg/d)	1.316 ± 0.68
Folate (ug/d)	177.1 ± 121.20
Niacin (mg/d)	12.83 ± 5.32
Vitamin C (mg/d)	79.82 ± 74.95
Vitamin A (R.E/d)	403.3 ± 277.80
Retinol (ug/d)	82.39 ± 49.32
Carotene (ug/d)	1810 ± 1533
Vitamin E (mg/d)	7.682 ± 4.12
Calcium (mg/d)	381.5 ± 213.90
Phosphorus (mg/d)	776.3 ± 305.70
Potassium (mg/d)	1813 ± 934.30
Iron (mg/d)	9.183 ± 4.66
Sodium (mg/d)	1982 ± 1160
Zinc (mg/d)	6.797 ± 2.73
Ash (mg/d)	12.74 ± 7.00

*Values are means and standard deviations*.

**Table 2:** General characteristics of participants at baseline.

	**All**
**Characteristics**	**(*n* = 66)**
Men; n (%)	29 (43.94)
Women; n (%)	37 (56.06)
Age (yr)	40.89 ± 9.94
Age group (yr)	
20–29; n (%)	7 (10.61)
30–39; n (%)	28 (42.42)
40–49; n (%)	19 (28.79)
50–59; n (%)	8 (12.12)
60–69; n (%)	4 (6.06)
Height (cm)	166.65 ± 7.59
Weight (kg)	64.86 ± 11.77
BMI (kg/m^2^)	23.22 ± 2.96

*Values are means and standard deviations*.

### Effect of *Saengshik* Consumption on Gut Microbial Diversity and Composition

**Figure 2:**
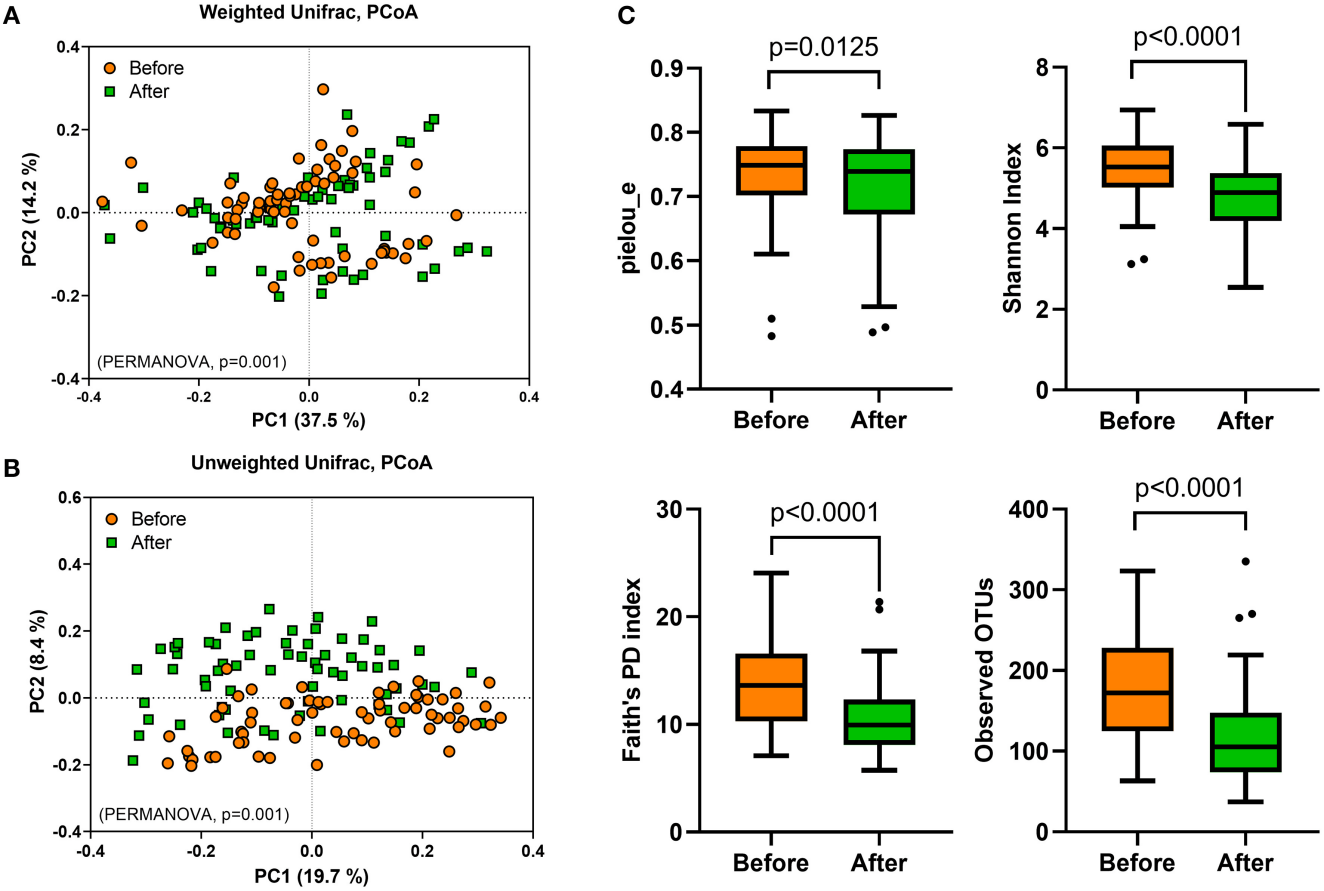
Gut microbial diversity is altered following *Saengshik* supplementation. The β-diversity of gut microbiota was compared using principal coordinates analysis plot based on **(A)** weighted and **(B)** unweighted UniFrac distances. A PERMANOVA test was used to evaluate differences in bacterial diversity between the two time points [before (orange; *n* = 66) and after (green; *n* = 66) intervention]. **(C)** Comparison of α-diversity in gut microbiota between the two time points [before (orange; *n* = 66) and after (green; *n* = 66) intervention]. Tukey's box-and-whisker plots show the median (horizontal line) and interquartile range (IQR) (box), with additional data within 1.5 IQR shown as whiskers. The Pielou's evenness, Shannon, Faith's PD, and observed OTU indices of the samples were statistically compared between the two time points using the Wilcoxon rank-sum test.

To evaluate the effect of *Saengshik* administration on gut microbiota profiles, we performed 16S rRNA sequencing analysis of stool samples collected at baseline and post-intervention. First, we evaluated β-diversity by calculating the weighted and unweighted unique fraction metric (UniFrac) distances, finding that the overall gut microbial community differed significantly between baseline and post-intervention (weighted *F* = 6.365, *P* = 0.001; unweighted *F* = 7.489, *P* = 0.001; PERMANOVA) (Figures 2A,B). To measure differences in diversity and evenness between baseline and post-intervention samples, we calculated Pielou's evenness (pielou_e), Shannon, Faith's phylogenetic diversity (Faith's PD), and observed OTU indices, which revealed significant decreases in α-diversity following intervention as compared with that at baseline (pielou_e, *P* = 0.0125; Shannon, *P* < 0.0001; Faith's PD, *P* < 0.0001; observed OTUs, *P* < 0.0001; Wilcoxon signed-rank test) (Figure 2C).

**Figure 3:**
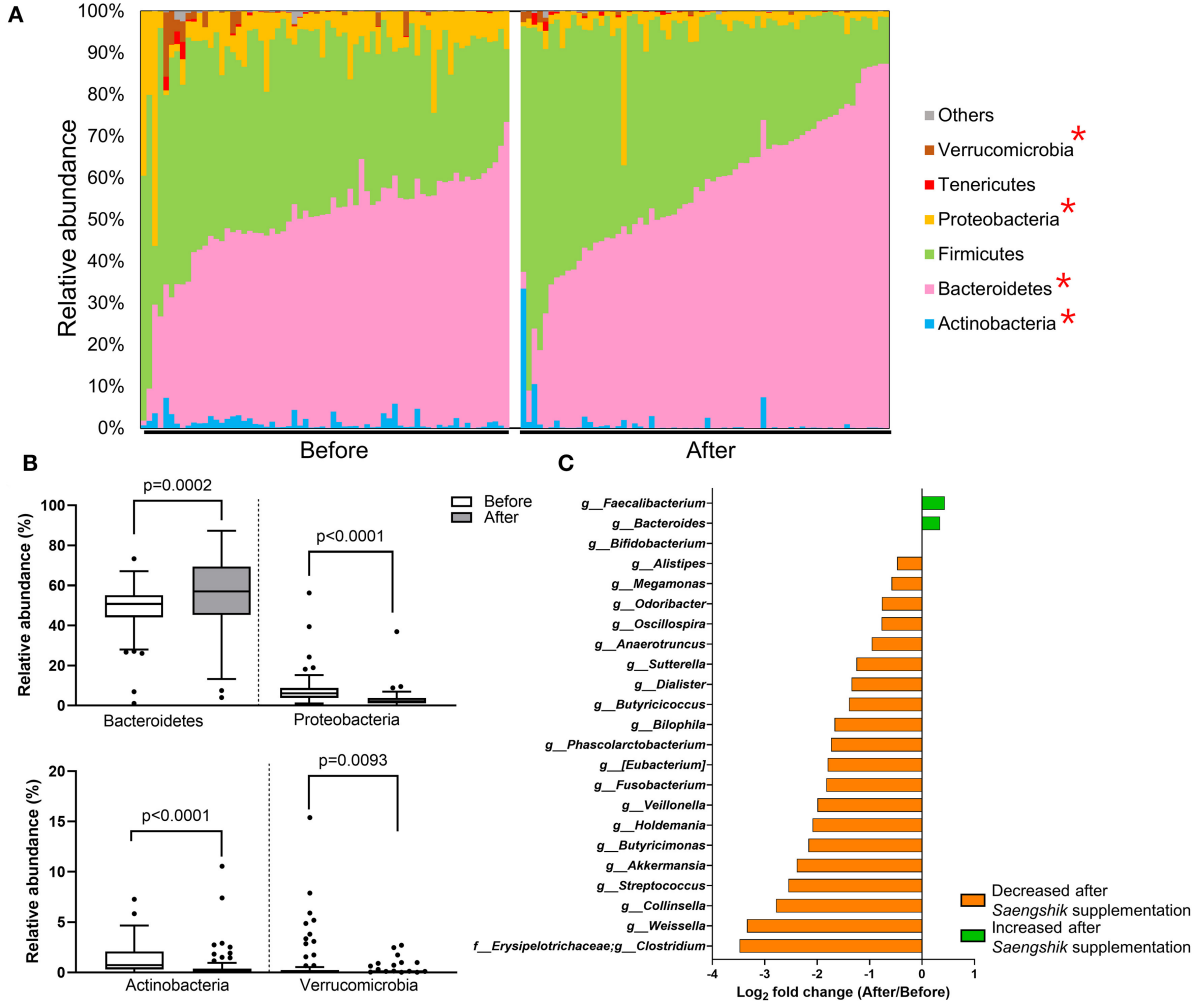
The relative abundance of bacterial taxa is affected by *Saengshik* intervention. **(A)** Distribution of gut microbial phyla before (*n* = 66) and after (*n* = 66) intervention. Each bar represents an individual participant. The proportion of the various taxa in each phylum is presented as a percentage of relative abundance with respect to total bacterial sequences. Different color schemes represent different phyla (blue: Actinobacteria, pink: Bacteroidetes, green: Firmicutes, yellow: Proteobacteria, red: Tenericutes, brown: Verrucomicrobia, gray: others). **(B)** Differences in the mean relative abundance of phyla before (*n* = 66) and after (*n* = 66) intervention. Tukey's box-and-whisker plots show the median (horizontal line) and interquartile range (IQR) (box), with additional data within 1.5 IQR shown as whiskers. The white and gray box plots represent the mean relative abundance of phyla before and after the intervention, respectively. **(C)** List of significantly changed genera according to genera found before (*n* = 66) and after (*n* = 66) intervention. Box plots of relative abundance show the fold change after vs. before intervention on the log_2_ scale. *P* < 0.01, Wilcoxon signed-rank test. The green and orange bars represent the bacterial genera that increased and decreased in abundance after *Saengshik* consumption, respectively.

To detect the specific bacterial taxa that were significantly affected by *Saengshik* consumption, we compared the composition of gut microbiota at the phylum and genus levels before and after intervention. Figure 3A shows the relative abundance (%) of each bacterial phylum of participants as different color bars. The distribution of *Bacteroidetes* increased after *Saengshik* consumption, whereas that of *Verrucomicrobia, Proteobacteria*, and *Actinobacteria* decreased, with statistically significant changes observed in the relative abundance of all phyla [*Bacteroidetes* (*P* = 0.0002), *Proteobacteria* (*P* < 0.0001), *Actinobacteria* (*P* < 0.0001), and *Verucomicrobia* (*P* = 0.0093)] (Figure 3B). At the genus level, we identified 23 genera showing significant differences in abundance between baseline and post-intervention (*P* < 0.01, Wilcoxon signed-rank test), with Figure 3C showing fold changes for each genus. The relative abundance of *Faecalibacterium, Bacteroides*, and *Bifidobacterium* significantly increased after *Saengshik* consumption, whereas that of *Alistipes, Megamnas, Odoribacter, Oscillospira, Anaerotruncus, Sutterella, Dialister, Butyricicoccus, Bilophila, Phascolarctobacterium, (Eubacterium), Fusobacterium, Veillonella, Holdemania, Butyricimonas, Akkermansia, Streptococcus, Collinsella, Weissella*, and *Clostridium* significantly decreased (Figure 3C). Collectively, these data demonstrate that daily intake of a plant-based food powder altered gut microbial communities and decreased gut microbial diversity. Furthermore, we identified specific bacterial phyla and genera that were significantly affected by *Saengshik* consumption.

### Effect of Individual Enterotype on Changes in the Gut Microbial Composition After *Saengshik* Consumption

**Figure 4:**
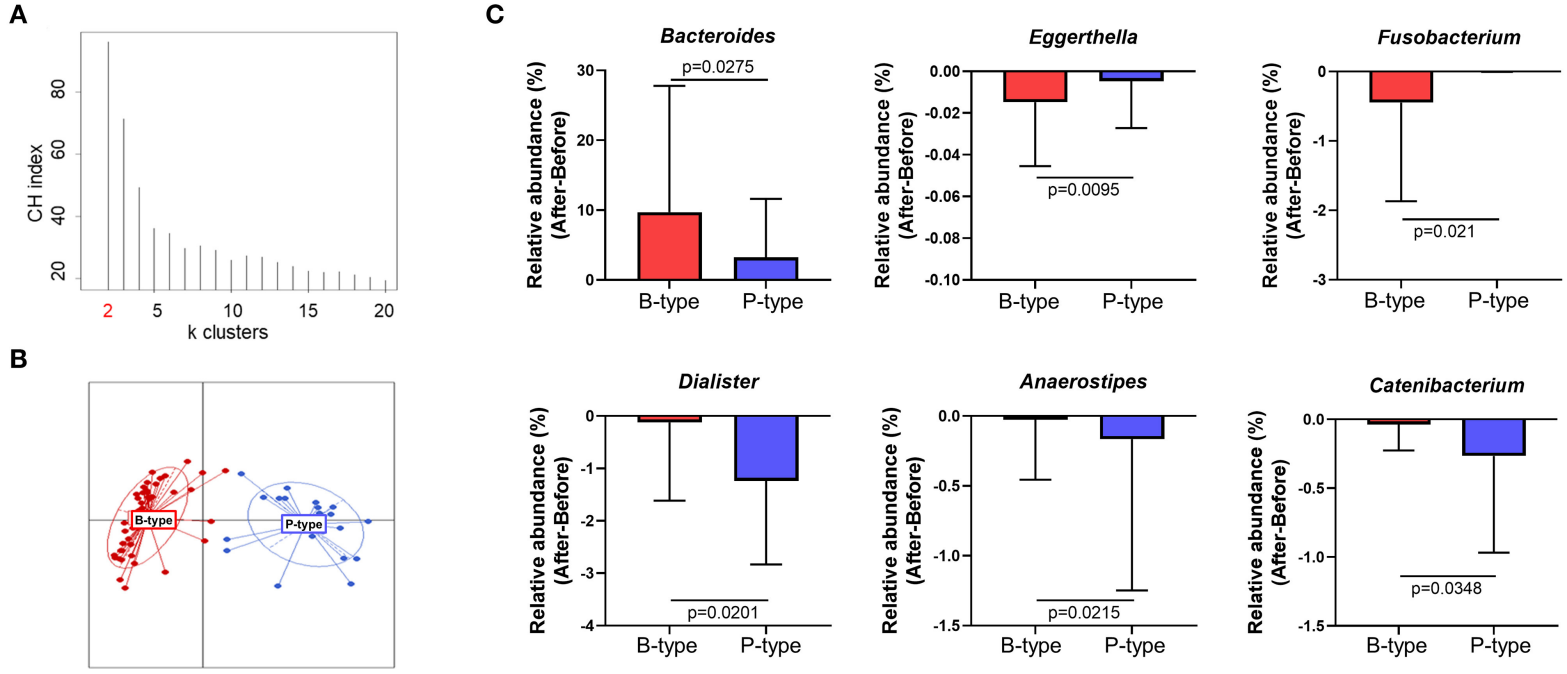
Changes in gut microbial composition after *Saengshik* supplementation according to enterotype. **(A)** Calinski–Harabasz (CH) indices for the number of potential clusters of samples. The x and y axes show the cluster number and CH index, respectively. The highest CH index refers to an optimal number of clusters. **(B)** Clustering based on the first two principal coordinates of the Jensen–Shannon distances of the relative abundance of the genus. Red color represents the *Bacteroides*-enriched enterotype (B-type; *n* = 43), and blue color represents the *Prevotella*-enriched enterotype (P-type; *n* = 23). Lines represent distances of points from centroids; ellipses show areas of highest density. **(C)** Changes in the relative abundance of significantly different gut bacterial genus between B-type (red; *n* = 43) and P-type (blue; *n* = 23) groups. The vertical axis shows the alternation of relative abundance, which is the delta value between the relative abundance (%) of samples before and after the intervention in each enterotype (After minus Before in P-type and B-type). *P*-values according to multiple *t*-tests were adjusted by the Benjamini–Hochberg false discovery rate (q < 0.05).

The human gut microbiome reportedly comprises two or three strains of major indigenous bacteria, which are referred to as the enterotype. Enterotypes reportedly reflect the unique gut microbial characteristics of individuals and are unaffected by body mass index (BMI), sex, or race. To verify whether enterotypes can affect compositional changes in gut microbiota after *Seangshik* consumption, we classified participant enterotypes and compared changes in gut microbiota among enterotypes. Following identification of a Calinski–Harabasz index of *k* = 2 as an optimal number of clusters, we confirmed that 66 participants were separated into two distinct clusters (Figures 4A,B), of which 43 belonged to the *Bacteroides*-rich enterotype (B-type) and 23 to the *Prevotella*-rich enterotype (P-type). The relative abundance of *Bacteroides* was significantly greater in B-type than that in P-type (*P* < 0.0001), whereas *Prevotella* was more abundant in P-type than in B-type (*P* < 0.0001) (Supplementary Figure 2). Additionally, we observed no statistically significant differences in age, height, weight, or BMI between individuals characterized as B- or P-type (all *P* > 0.05) (Supplementary Table 3). We then examined whether changes in the relative abundance of genera Figure 3C differed significantly according to enterotype before and after *Saengshik* intake. Figure 4C shows that the relative abundance of *Bacteroides* (*P* = 0.028) was significantly higher in B-type individuals relative to P-type individuals after *Seangshik* consumption, whereas the relative abundance of *Eggerthella* (*P* = 0.010) and *Fusobacterium* (*P* = 0.021) decreased significantly decreased in B-type relative to P-type individuals. However, *Dialister* (*P* = 0.020), *Anaerostipes* (*P* = 0.022), and *Catenibacterium* (*P* = 0.035) decreased significantly in P-type relative to B-type individuals after *Seangshik* consumption (Figure 4C). These observations suggest that enterotypes affected the response of gut microbial composition to *Saengshik* consumption.

### Effect of Habitual Dietary Patterns on Changes in Gut Microbial Composition After *Saengshik* Consumption

**Figure 5:**
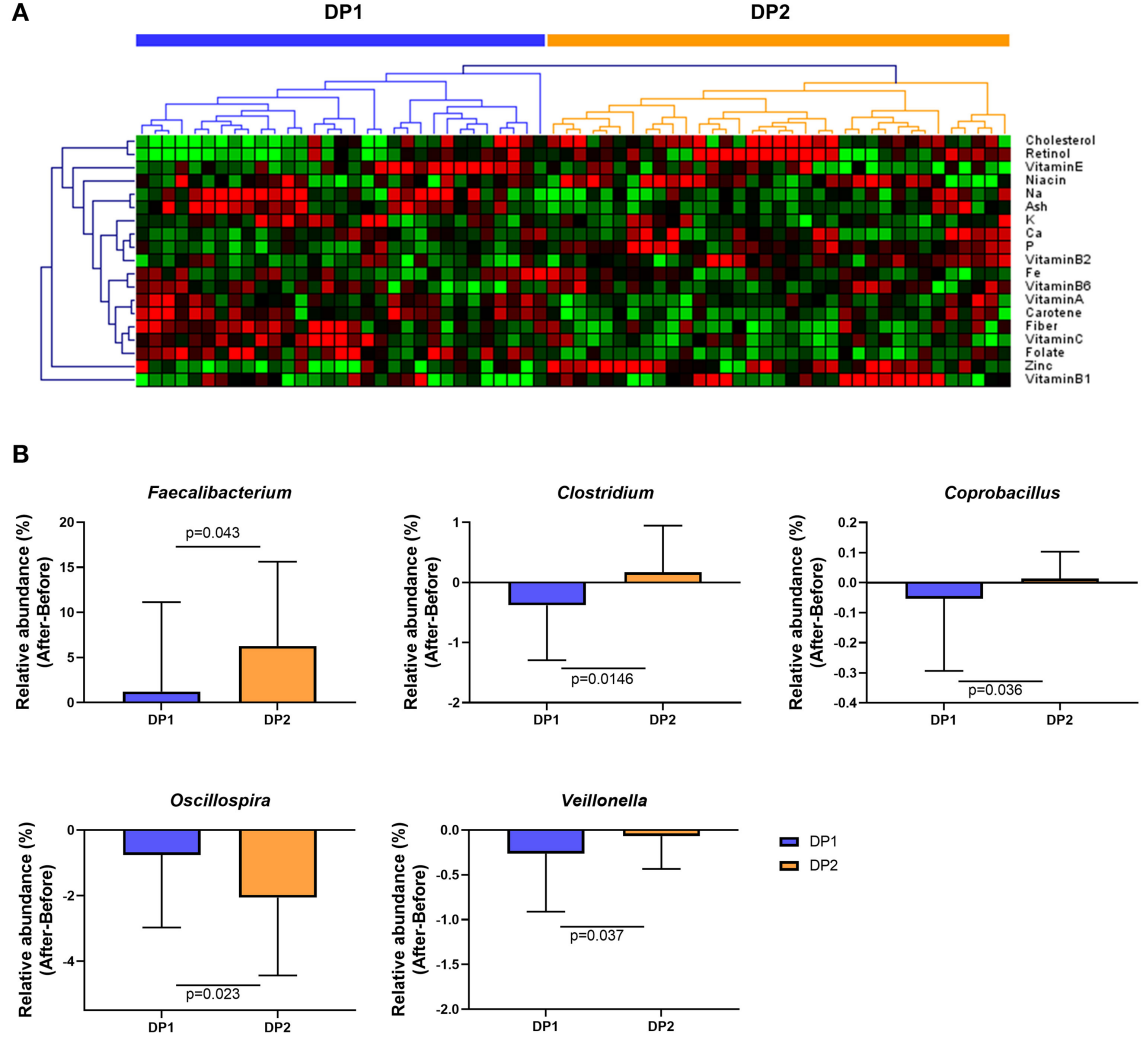
Changes in gut microbial composition after *Saengshik* supplementation according to dietary-pattern clusters. **(A)** Hierarchical clustering heatmap of nutrient-consumption data. Results of unsupervised cluster analysis revealing two clusters based on nutrient intake of participants. Each column shows the nutrient-intake pattern of individuals in the DP1 (*n* = 31) and DP2 (*n* = 35) groups. The amount of each nutrient in individual samples is expressed as a relative value obtained by the auto-scaling method and represented by color, where red and green indicate high and low concentrations of nutrients, respectively. Values were measured by Euclidean distance with an average linkage clustering algorithm. **(B)** Changes in the relative abundance of significantly different gut bacterial genera between the DP1 (blue; *n* = 31) and DP2 (orange; *n* = 35) groups. The vertical axis shows the alternation of relative abundance, which is the delta value between the relative abundance (%) of samples before and after the intervention in each dietary pattern group (After minus Before in DP1 and DP2). *P*-values according to multiple *t*-tests were adjusted by the Benjamini–Hochberg false discovery rate (q < 0.05).

To investigate whether habitual dietary patterns affect changes in gut microbial composition after *Saengshik* consumption, we compared changes in gut microbiota among subgroups classified by habitual dietary patterns. Figure 5A shows the results of clustering each subject based on the amount of micronutrient intake per 1000 kcal/day at baseline and according to FFQ data. We specified these two subgroups as dietary pattern 1 (DP1; *n* = 31) and DP2 (*n* = 35). There were no significant differences in age, height, weight, or BMI between DP1and DP2 (all *P* > 0.05) (Supplementary Table 4). Those in DP1consumed higher amounts of carbohydrates, fiber, vitamin C, and vitamin E, whereas those in the DP2consumed higher amounts of protein, fat, cholesterol, vitamin B_1_, retinol, carotene, and zinc (all *P* < 0.05) (Supplementary Table 5). We then determined whether changes in the relative abundance of genera (Figure 3C) differed significantly according to individual dietary pattern in each subgroup and compared these changes before and after Saengshik intake. The results showed that the relative abundance of *Faecalibacterium* (*P* = 0.043), *Clostridium* (*P* = 0.015), and *Coprobacillus* (*P* = 0.036) was significantly higher in DP2 than in DP1 after *Seangshik* consumption (Figure 5B). Moreover, the relative abundance of *Oscillospira* decreased significantly in DP2 as compared with that in DP1 (*P* = 0.023); however, *Veillonella* decreased significantly in DP1 as compared with that in DP2 (*P* = 0.037) (Figure 5B). These results indicate that compositional changes in gut microbiota after *Saengshik* consumption were differentially affected by individual dietary patterns.

### Collective Effect of Individual Enterotype and Habitual Dietary Patterns on Changes in Specific Genera Compositions

**Figure 6:**
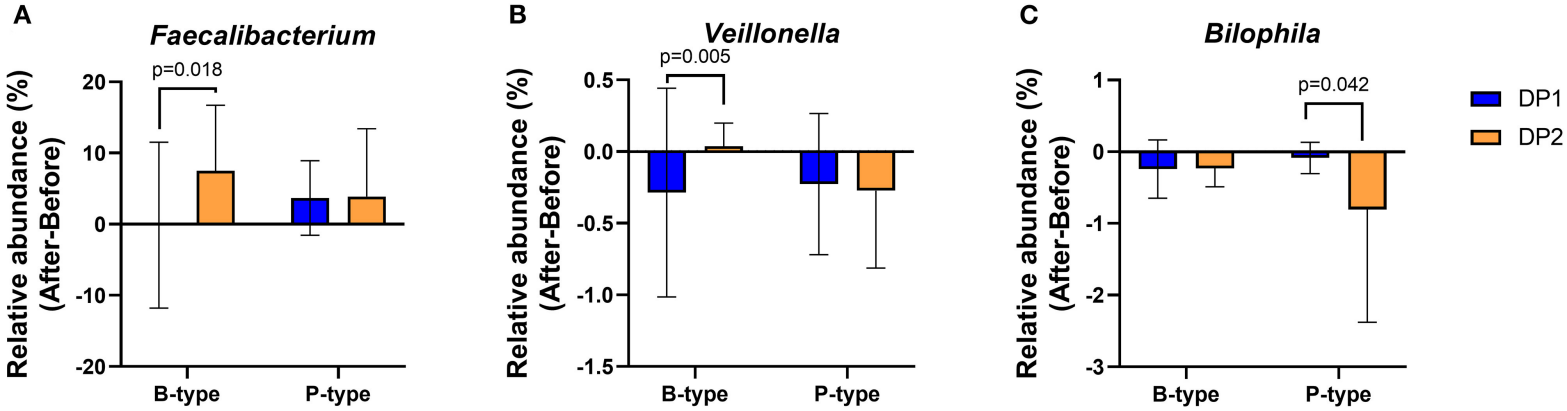
Changes in gut microbial composition after *Saengshik* supplementation according to enterotype and dietary-pattern clusters. Changes in the relative abundance of **(A)** *Faecalibacterium*, **(B)** *Veillonella*, and **(C)** *Bilophila* according to enterotype and habitual dietary-pattern group. The vertical axis shows the alternation of relative abundance, which is the delta value between the relative abundance (%) of samples before and after the intervention in each dietary pattern and enterotype group (After minus Before in B-type with DP1, B-type with DP2, P-type with DP1, and P-type with DP2). *P*-values according to multiple *t*-tests were adjusted by the Benjamini–Hochberg false discovery rate (q < 0.05). B-type: DP1 (*n* = 20) and DP2 (*n* = 23). P-type: DP1 (*n* = 11) and DP2 (*n* = 12).

To determine whether enterotype and habitual dietary patterns can collectively influence changes in gut microbial genera after *Saengshik* consumption, we compared changes in 23 genera (Figure 3C) between subjects with different dietary patterns in the same enterotype. In the B-type, 20 and 23 individuals belonged to the DP1 and DP2 clusters, respectively, whereas in the P-type, 11 and 12 individuals belonged to each respective cluster (Supplementary Figure 3). Notably, three of 29 genera were affected by enterotypes and habitual dietary patterns. After intervention and among B-type individuals, the relative abundance of *Faecalibacterium* (*P* = 0.018, Mann–Whitney *U* test) and *Veillonella* (*P* = 0.005, Mann–Whitney *U* test) increased in DP2 individuals but not in DP1 individuals. There was no difference in changes in the abundance of these two genera between DP1 and DP2 P-type individuals (Figures 6A,B). Among P-type individuals, DP2 showed a larger decrease in the relative abundance of *Bilophila* than DP1 individuals (*P* = 0.042, Mann–Whitney *U* test) (Figure 6C). Collectively, these results indicate that *Saengshik* consumption changed gut microbial communities, which were influenced by not only individual habitual dietary patterns but also enterotype.

## Discussion

This study provides new insights into the effects of *Saengshik*, a plant-based commercial powder, in altering the composition of fecal microbiota in healthy adults. The results confirmed the hypothesis that *Saengshik* can directly alter specific bacterial taxa, and that these changes depend on individual baseline gut microbiome and diet patterns. Moreover, bacterial diversity after intervention was significantly lower than that before, and importantly, the relative abundance of *Faecalibacterium, Veillonella*, and *Bilophila* was differentially influenced by a combination of baseline gut microbiota and diet patterns. Thus, these findings suggest that baseline gut microbiota and diet patterns can promote modifications in gut microbiota composition following supplementation.

### *Saengshik* Consumption and Gut Microbial Diversity

Previous studies revealed that plant-rich diets are related to increased bacterial diversity because of their capability to provide a source of microbiota-accessible carbohydrates (30, 31). Compared with plant-rich diets, a Western-style diet is characterized by high intake of saturated fat and simple carbohydrates, whereas low intake of fiber is associated with reduced bacterial diversity (32). Additionally, microbial diversity is inversely associated with chronic diseases and metabolic dysfunction (33, 34). Therefore, we expected that *Saengshik* intake would improve gut microbial diversity; however, contrary to our expectation, we observed significant decreases in gut microbial diversity after 8 weeks of *Saengshik* consumption relative to that at baseline. Given that the average daily fiber intake of Korean adults is 24.3 g (35), we speculated that the amount of fiber contained in the product used in this study (Supplementary Table 2) would not contribute to increasing the diversity of intestinal microbes. Multiple *in vivo* and *in vitro* studies have revealed that phenolic compounds and flavonoids exhibit antibacterial activity against several pathogenic bacteria (36, 37). However, discrepancies in the antibacterial properties of even the same phenolic compounds have been reported among bacterial strains (38). Therefore, the antimicrobial activity of various phenolic compounds and flavonoids present in *Saengshik* might selectively affect different strains of gut microbiota, which might have contributed to the reduced diversity observed in the present study. Another possibility is that the various food components consumed as complex combinations in daily meals during the intervention period might have had an interactive effect (39), although this factor was not ruled out in the present study. Furthermore, the present study was conducted in healthy adults who already showed relatively high gut microbial diversity. The possibility of individuals with low bacterial diversity being more responsive to *Saengshik* intake should be considered. Additionally, some interventions or cross-sectional studies report contradictory findings, including cases in which changes in diversity were not observed (40–42) or those in which diversity decreased after a plant-based diet (43, 44). Thus, to confirm the effect of *Saengshik* intake on gut microbial diversity, well-controlled and designed clinical trials are needed for individuals with decreased microbial diversity.

### The Physiological Role of Specific Gut Microbial Taxa Which Changed by *Saengshik* Intake

We found that the population of *Faecalibacterium* increased significantly after *Saengshik* consumption, which might be related to positive effects on health. This result is consistent with the correlation between *Faecalibacterium* and plant-food intake revealed reported in previous studies (40, 45). *Faecalibacterium* can utilize non-digestible carbohydrates (dietary fiber) from host mucus and concomitantly produce butyrate, which is associated with several beneficial health properties (46). This might be a factor related to the beneficial effects of *Saengshik*, including its anti-obesity and anti-inflammatory effects. Moreover, recent studies demonstrated an inverse association between *Faecalibacterium* and the risk of diseases, such as obesity (47), inflammatory colitis (48), and diabetes (49). In the present study, we also showed that the abundance of *Akkermansia* and *Collinsella* decreased after *Seangshik* consumption. *Akkermansia* exhibits probiotic potential that has been linked to maintaining intestinal barrier integrity, anti-inflammatory properties, and immune function (50). By contrast, other studies reported that *Akkermansia* spp. exacerbated gut inflammation in an animal infection model based on their ability to degrade mucins (51). Furthermore, elevated *Collinsella* abundance is reportedly positively associated with increased levels of insulin, cholesterol, and triglycerides (52) and negatively correlated with vegetable intake (53, 54). In line with previous studies, the present results suggest that a lower abundance of *Collinsella* might be mediated by *Saengshik* consumption. Nevertheless, the reduced abundance of beneficial microbes, including *Butyricimonas, Megamonas, Phascolarctobacterium*, and *Butirycicoccus*, should be verified through further studies. Collectively, although it is difficult to conclude that the changes in intestinal microflora caused by *Saengshik* would have a positive effect on host health, these findings show a possible link between gut microbiota, *Saengshik* consumption, and physiological properties.

### The Relationship Between Nutrients Contained in *Saengshik* and Intestinal Microflora

*Saengshik* contains many micronutrients and compounds, including phenolics and flavonoids. Studies suggest that the gut microbiome-altering effects of *Saengshik* might originate from a variety of these ingredients. Indeed, several clinical trials report that polyphenol consumption can modulate gut microbial composition, anthropometric variables, and clinical markers (55). In the present study, we found that *Saengshik* consumption increased the abundance of *Bacteroidetes, Bifidobacterium*, and *Bacteroides*. Consistent with these findings, an intervention study reported increases in these microbes following intake of polyphenol-rich foods, such as red wine, freeze-dried cranberry powder, fruits, and vegetables (15, 56–58). Moreover, *Bacteroides* and *Bifidobacterium* are negatively correlated with various clinical markers, such as tumor necrosis factor-α, cholesterol, blood pressure, and lipopolysaccharide (55) levels. These results support our findings that *Saengshik* might be involved in beneficial changes in biological markers. In addition to being a good source of flavonoids and phenolic compounds, *Saengshik* is high in folate. The serving size of *Saengshik* (40 g) used in this study contained folate at a level of 83% of the recommended dietary allowance for Korean adults (400 μg) (59); therefore, the benefit of a sufficient folate intake could be expected. Mammals cannot synthesize their own folate *de novo*; therefore, they need to obtain folate via the diet and gut microbes, such as *Lactobacillus* and *Bifidobacterium* (60). Mice with folate deficiency reportedly show a significant increase in the *Firmicutes*:*Bacteroidetes* ratio (61), and cross-talk between gut microbiota and folate can modulate the regeneration of intestinal epithelial cells through stem cells by epigenetic modification (62). Although it remains unclear whether dietary folate intake is directly attributable to the composition and metabolism of gut microbiota (63), the consumption of sufficient folate (i.e., >3-fold greater than the habitual intake of the study participants) might contribute to microbial changes.

### Effect of Individual Enterotype and Dietary Pattern on Changes in Intestinal Microbiota Caused by *Saengshik* Consumption

A notable finding in the present study was the inter-individual variability in response to the intervention, which was similar to results from clinical trials investigating the effect of diet on gut microbiota. Here, we showed that B-type individuals in the DP2 group showed a stronger increase in *Faecalibacterium* levels than those with DP1 group. *Faecalibacterium* reportedly plays a crucial role in maintaining human health owing to its production of anti-inflammatory metabolites or short-chain fatty acids. Indeed, *Faecalibacterium* is less abundant in patients with many diseases relative to healthy populations (47–49). Additionally, we showed that *Veillonella* abundance increased in individuals with a B-type enterotype and those with unhealthy dietary habits, whereas it decreased in individuals belonging to the B-type and DP1 groups. A recent study on healthy non-obese adults reported that increased abundance of *Veillonella* was negatively associated with saturated fatty acid consumption and body fat percentage (64). Furthermore, *Veillonella* abundance increases in women with high fiber intake, with its abundance associated with improved insulin sensitivity (65). Based on these findings, we suggest that adults with “less healthy” diet patterns and a *Bacteroides*-dominant enterotype might experience greater *Saengshik*-mediated positive health benefits. Moreover, these results suggest that further investigation of the role of habitual dietary patterns or enterotypes in evaluating dietary intervention is warranted.

### Study Limitations

This study has several limitations. First, given to the descriptive nature of the study and the absence of related clinical outcomes, we cannot provide insights into the beneficial properties of changed bacterial taxa related to *Saengshik* consumption. Second, the study lacked use of a placebo or blank control and presented a small sample size. Third, given that we did not collect 3-day or 24-h diet records of participants, we cannot completely rule out the possibility that differences in the usual dietary intake before and after intervention could have affected the outcomes. Finally, pre-selected participants with dysbiosis or low bacterial diversity rather than heathy individuals might be more suitable for identifying the effect of *Saengshik* on gut microbiota. Therefore, well-designed and controlled clinical trials with a larger sample size are necessary to determine the biological effect of *Saengshik* on the gut microbial community.

Despite these limitations, this represents the first study providing evidence that *Saengshik* consumption by healthy adults can influence gut microbial composition and diversity. Furthermore, the observed compositional changes were influenced by not only baseline gut microbial composition (enterotype) but also habitual dietary intake. We believe that this study underlines the need to investigate both baseline gut microbiota composition and diet patterns to allow the design of personalized interventions for diet-induced changes in gut microbiota and human health.

## Conclusion

In summary, we showed that *Saengshik* consumption altered gut microbial composition and diversity. The results demonstrated that in healthy Korean adults, the effect of *Saengshik* intake on gut microbial composition was dependent on individual enterotype and dietary patterns at baseline. Although the precise mechanisms responsible for this outcome remain unclear, their identification could potentially yield new approaches for maintaining overall health linked to gut microbiota.

## Data Availability Statement

The datasets presented in this study can be found in online repositories. The names of the repository/repositories and accession number(s) can be found at: https://dataview.ncbi.nlm.nih.gov/object/PRJNA747737.

## Ethics Statement

The studies involving human participants were reviewed and approved by Institutional Review Board (IRB) of Theragen Bio (IRB No. 700062-20180905-JR-005-01). The patients/participants provided their written informed consent to participate in this study.

## Author Contributions

Y-DN and YJA conceived and designed the project. J-HK and MHP provided an experimental supplement and technical support. MYL and YJA collected the samples and managed the intervention study. J-HS, W-HC, and SPH conducted data analysis. J-HS, W-HC, and Y-DN wrote the manuscript. All authors read and approved the final version of the manuscript.

## Funding

This research was supported by the Main Research Program (E0170600-05) of the Korea Food Research Institute (KFRI) funded by the Ministry of Science and ICT.

## Conflict of Interest

YJA was employed by the company Theragen Bio Co., Ltd. MHP and J-HK were employed by the company Erom Co. Ltd. The remaining authors declare that the research was conducted in the absence of any commercial or financial relationships that could be construed as a potential conflict of interest. Furthermore, this study did not receive any funding from Theragen Bio Co. or Erom Co. Ltd.

## Publisher's Note

All claims expressed in this article are solely those of the authors and do not necessarily represent those of their affiliated organizations, or those of the publisher, the editors and the reviewers. Any product that may be evaluated in this article, or claim that may be made by its manufacturer, is not guaranteed or endorsed by the publisher.
