# Supplementary material for: Effect of Saengshik Supplementation on the Gut Microbial Composition of Healthy Korean Adults: A Single-Group Pilot Study
Source: Front Nutr. 2021 Oct 22;8:743620. doi: 10.3389/fnut.2021.743620 (PMC8568882; doi:10.3389/fnut.2021.743620)
Supplement: Supplementary file 1 [file Data_Sheet_1.docx]

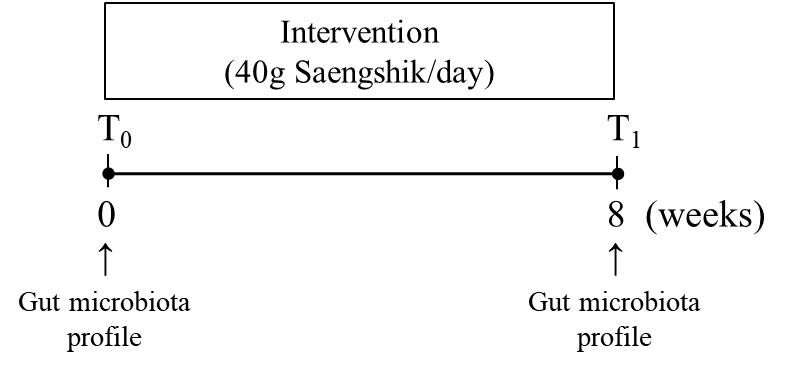


**Supplementary Figure 1.** Study design.


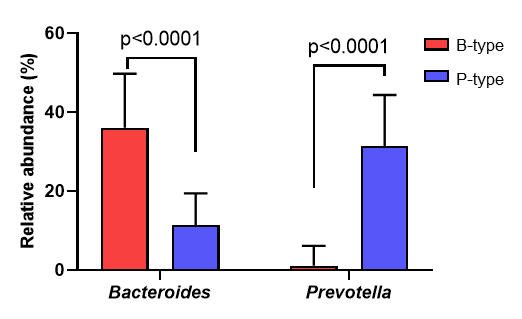


**Supplementary Figure 2**. Relative abundances of *Bacteroides* and *Prevotella* in each enterotype. B-type, *Bacteroides*-enriched enterotype; P-type, *Prevotella*-enriched enterotype.


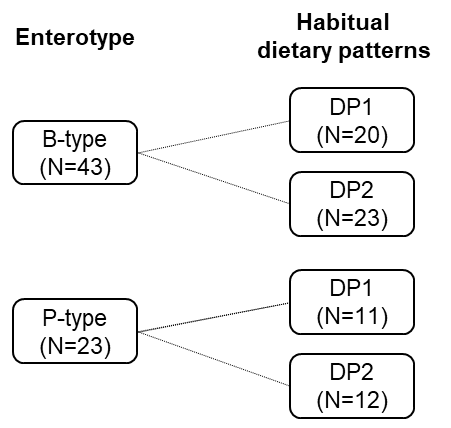


**Supplementary Figure 3.** The numbers of participants assigned to each enterotype and dietary pattern cluster. B-type, *Bacteroides*-enriched enterotype; P-type, *Prevotella*-enriched enterotype; HDP, healthy dietary pattern group; LHDP, less healthy dietary pattern group.

**Supplementary Table 1.** Materials of *Saengshik*

| Classification | Materials |
| --- | --- |
| Grains | Brown rice, Black rice, Barley, Ganoderma lucidum Rice, Waxy Brown Rice, Waxy millet, Sorghum, Proso millet, Adlay, Black sesame |
| Beans | Soybean, White soybean, Red bean, Small black bean (*Rhynchosia nulubilis*) |
| Vegetables | Kale, Carrot, Pumpkin, Spirulina, Cabbage, Burdock, Radish leaves, Broccoli, White radish, Chinese chive, Barley sprout, Tomato, *Angelica keiskei,* Potato |
| Fruits | Yuzu, Luo han guo (*Siraitia grosvenorii)* |
| Mushroom | Oyster mushroom, Ganoderma lucidum mushroom, Shiitake mushroom |
| Sea weed | Laver, Sea mustard, Sea tangle, Sea lettuce |
| The others | Rice bran, Mugwort, Small water dropwort (*Oennathe javanica)* |

**Supplementary Table 2.** Nutrients ingredient of *Saengshik*

| Nutrients | Amount per gram | Amount per serving size (40g) |
| --- | --- | --- |
| Energy (kcal) | 4 | 160 |
| Carbohydrates (g) | 0.825 | 33 |
| Protein (g) | 0.125 | 5 |
| Fat (g) | 0.038 | 1.52 |
| Cholesterol (mg) | 0 | 0 |
| Fiber (g) | 0.005 | 0.2 |
| Sodium (mg) | 1.625 | 65 |
| Potassium (mg) | 4.270 | 170.8 |
| Calcium (mg) | 2.746 | 109.84 |
| Magnessium (mg) | 1.736 | 69.44 |
| Phosphorus (mg) | 3.510 | 140.4 |
| Beta-carotene(mg) | 0.003 | 0.12 |
| VitaminE(mg) | 0.016 | 0.64 |
| VitaminC(mg) | 0.139 | 5.56 |
| Folate (ug) | 8.248 | 329.92 |
| Vitamin B1 (mg) | 0.006 | 0.24 |
| Niacine (mg) | 0.136 | 5.44 |
| VitaminB2 (mg) | 0.002 | 0.08 |
| Total phenolic compound (ug) | 1005.250 | 40210 |
| Total flavonoid (ug) | 231.910 | 9276.4 |

**Supplementary Table 3.** General characteristics of subjects according to enterotype

| Characteristics | B-type  (n=43) | P-type  (n=23) | P-value^†^ |
| --- | --- | --- | --- |
| Men/Women (n/n) | 19/24 | 10/13 |  |
| Age (yr) | 41.07 ± 10.19 | 40.57 ± 9.67 | 0.8460 |
| Height (cm) | 166.91 ± 7.39 | 166.17 ± 8.11 | 0.7117 |
| Weight (kg) | 66.21 ± 11.87 | 62.35 ± 11.41 | 0.2065 |
| BMI (kg/m^2^) | 23.65 ± 3.08 | 22.42 ± 2.58 | 0.1076 |

Values are mean ± SD. ^†^ P values based on unpaired t test. B-type, *Bacteroides*-enriched enterotype; P-type, *Prevotella*-enriched enterotype.

**Supplementary Table 4**. General characteristics of subjects according to dietary pattern clusters

| Characteristics | DP1  (n=31) | DP2  (n=35) | P-value^†^ |
| --- | --- | --- | --- |
| Men/Women (n/n) | 14/17 | 15/20 |  |
| Age (yr) | 42.42 ± 9.31 | 39.54 ± 10.41 | 0.2437 |
| Height (cm) | 166.52 ± 7.84 | 166.77 ± 7.48 | 0.8928 |
| Weight (kg) | 65.00 ± 11.67 | 64.74 ± 12.03 | 0.9302 |
| BMI (kg/m^2^) | 23.30 ± 2.76 | 23.15 ± 3.16 | 0.8381 |

Values are mean ± SD. ^†^P values based on unpaired t test. DP1, dietary pattern 1 group; DP2, dietary pattern 2 group.

**Supplementary Table 5.** The nutrients intake per 1000 kcal in participants at baseline

| Nutrients^1^ | DP1 | | DP2 | | P-value^2^ |
| --- | --- | --- | --- | --- | --- |
|  | (n=31) | | (n=35) | |  |
|  | Mean | SD | Mean | SD |  |
| Carbohydrate (g) | 165.98 | 18.35 | 151.12 | 21.85 | 0.004 |
| Protein (g) | 34.61 | 6.33 | 38.56 | 8.27 | 0.035 |
| Fat (g) | 21.45 | 6.49 | 25.77 | 7.50 | 0.016 |
| Fiber (g) | 3.13 | 1.14 | 2.30 | 0.92 | 0.002 |
| Cholesterol (mg) | 113.32 | 52.59 | 169.68 | 88.51 | 0.003 |
| VitaminB1(mg) | 0.63 | 0.12 | 0.71 | 0.17 | 0.033 |
| VitaminB2(mg) | 0.53 | 0.14 | 0.58 | 0.17 | 0.209 |
| VitaminB6(mg) | 0.85 | 0.21 | 0.84 | 0.21 | 0.873 |
| Folate (ug) | 130.07 | 45.45 | 95.10 | 40.82 | 0.002 |
| Niacin (mg) | 8.22 | 1.84 | 8.66 | 2.01 | 0.358 |
| VitaminC (mg) | 60.99 | 34.40 | 39.96 | 20.97 | 0.003 |
| VitaminA (R.E) | 299.63 | 170.28 | 238.80 | 128.10 | 0.104 |
| Retinol (ug) | 43.53 | 20.17 | 69.41 | 38.55 | 0.001 |
| Carotene (ug) | 1478.67 | 951.55 | 927.91 | 618.34 | 0.006 |
| VitaminE (mg) | 5.52 | 1.69 | 4.53 | 1.55 | 0.016 |
| Calcium (mg) | 248.18 | 100.80 | 258.83 | 161.96 | 0.753 |
| Phosphorus (mg) | 482.48 | 96.91 | 540.18 | 143.70 | 0.064 |
| Potassium (mg) | 1247.89 | 392.59 | 1136.65 | 384.02 | 0.249 |
| Sodium (mg) | 1464.34 | 591.82 | 1159.38 | 595.72 | 0.041 |
| Iron (mg) | 6.28 | 2.15 | 5.77 | 1.62 | 0.275 |
| Zinc (mg) | 4.11 | 0.70 | 4.73 | 0.81 | 0.002 |
| Ash (mg) | 9.28 | 3.78 | 7.51 | 2.67 | 0.030 |

^1^Nutrient density per 1000kcal; ^2^P values based on unpaired t test. DP1, dietary pattern 1 group; DP2, dietary pattern 2 group.
